# Childhood opportunity and appropriate use of child safety restraints in motor vehicle collisions

**DOI:** 10.1136/wjps-2023-000703

**Published:** 2024-04-02

**Authors:** Stephanie Y Chen, Iris Garcia, Shadassa Ourshalimian, Chantel Lowery, Pradip P Chaudhari, Ryan G Spurrier

**Affiliations:** 1 Pediatric Surgery, Children's Hospital Los Angeles, Los Angeles, California, USA; 2 Injury Prevention Program, Children's Hospital Los Angeles, Los Angeles, California, USA; 3 Emergency and Transport Medicine, Children's Hospital Los Angeles, Los Angeles, California, USA; 4 Pediatrics, University of Southern California Keck School of Medicine, Los Angeles, California, USA; 5 Surgery, University of Southern California Keck School of Medicine, Los Angeles, California, USA

**Keywords:** Pediatrics, Emergency Service, Hospital

## Abstract

**Objectives:**

Safety restraints reduce injuries from motor vehicle collisions (MVCs) but are often improperly applied or not used. The Childhood Opportunity Index (COI) reflects social determinants of health and its study in pediatric trauma is limited. We hypothesized that MVC patients from low-opportunity neighborhoods are less likely to be appropriately restrained.

**Methods:**

A retrospective cross-sectional study was performed on children/adolescents ≤18 years old in MVCs between January 1, 2011 and December 31, 2021. Patients were identified from the Children’s Hospital Los Angeles trauma registry. The outcome was safety restraint use (appropriately restrained, not appropriately restrained). COI levels by home zip codes were stratified as very low, low, moderate, high, and very high. Multivariable regression controlling for age identified factors associated with safety restraint use.

**Results:**

Of 337 patients, 73.9% were appropriately restrained and 26.1% were not appropriately restrained. Compared with appropriately restrained patients, more not appropriately restrained patients were from low-COI (26.1% vs 20.9%), high-COI (14.8% vs 10.8%) and very high-COI (10.2% vs 3.6%) neighborhoods. Multivariable analysis demonstrated no significant associations in appropriate restraint use and COI. There was a non-significant trend that children/adolescents from moderate-COI neighborhoods were more likely than those from very low-COI neighborhoods to be appropriately restrained (OR=1.82, 95% CI 0.78, 4.28).

**Conclusion:**

Injury prevention initiatives focused on safety restraints should target families of children from all neighborhood types.

**Level of evidence:**

III.

**WHAT IS ALREADY KNOWN ON THIS TOPIC**

- Child safety restraints, when used properly, reduce injury and death from motor vehicle collisions. Disparities exist in pediatric trauma patients and may be explained more comprehensively by the Childhood Opportunity Index. There are limited studies that use the Childhood Opportunity Index in pediatric trauma.

**WHAT THIS STUDY ADDS**

- Many children in motor vehicle collisions are found to not be appropriately restrained. There is no significant association between appropriate safety restraint use and Childhood Opportunity Index.

**HOW THIS STUDY MIGHT AFFECT RESEARCH, PRACTICE OR POLICY**

- The findings of this study highlight that families from all neighborhood types may benefit from targeted injury prevention education regarding motor vehicle child safety restraints. This study also introduces the use of the Childhood Opportunity Index to investigate disparities among pediatric trauma patients.

## Introduction

Motor vehicle collisions (MVCs) are one of the leading causes of morbidity and mortality in children.^1–3^ Used properly, child safety restraints substantially reduce injury severity and risk of death in children in MVCs.^4 5^ Mortality from MVC in the USA is significantly associated with children who are either unrestrained or inappropriately restrained.^6–8^ Despite this, child safety restraints are often unused or improperly used, with recent literature reporting more than 50% of children were not properly restrained.^7 9 10^ Disparities in pediatric trauma have been broadly described, including in the context of MVCs and the use of child safety restraints.^5 11^ For example, Rangel *et al* previously demonstrated racial and financial disparities in child safety restraint use, with black children and children with public health insurance being less likely to be properly restrained compared with white children and those with commercial insurance.^12^

The Childhood Opportunity Index (COI) 2.0 is a multidimensional metric reflecting neighborhood resources conducive to healthy childhood development and represents a comprehensive method of describing neighborhood socioeconomic factors that influence child health outcomes.^13 14^ The COI has been used to investigate disparities in social determinants of health in general pediatric literature and, more recently, pediatric surgery.^15^ To date, there are limited studies that specifically use the COI to investigate disparities in pediatric trauma.

Therefore, our objective was to determine the association between appropriate restraint use and COI level for children in MVCs. We hypothesized that MVC patients from neighborhoods with low COI are less likely to be restrained or properly restrained.

## Methods

### Design, data source, and study population

An institutional review board-approved retrospective cross-sectional study was performed on all children and adolescents aged ≤18 years who presented to a free-standing children’s hospital and level 1 American College of Surgeons-verified pediatric trauma center in Los Angeles, California, following an MVC between January 1, 2011 and December 31, 2021. Patients were identified using our internal trauma registry, which captures all trauma activations at our institution. Data are entered into the trauma registry by trained trauma registrars following a standardized protocol. Exclusion criteria included mechanisms outside of MVCs (auto vs pedestrian, fall), in utero at time of MVC, and riding sports vehicles (all-terrain vehicles, golf carts), carnival rides, or public transportation where safety restraints are not universally available. Patients with missing insurance or zip code data due to residence outside of the USA were excluded.

The trauma database provided restraint data collected from the initial history and physical or emergency medical services (EMS) reports. Other variables collected from the trauma database included age, weight, height, race/ethnicity (as defined by medical record documentation), mechanism of injury, residential address including zip code, Injury Severity Score (ISS), disposition from the emergency department (ED) or location of admission, and insurance. Individual patient electronic medical records were reviewed to verify primary language as documented by healthcare personnel or based on the language in which education materials and consent forms were created.

### Outcome

The primary outcome was appropriate restraint use. The electronic medical records of patients categorized as non-restrained were individually reviewed to confirm that no restraint was used. Patients were stratified based on safety restraint use (non-restrained, car seat, child booster, lap belt, seat belt) and whether they were appropriately restrained or not appropriately restrained. Not appropriately restrained patients included those who were non-restrained or improperly restrained, such as using the wrong type of restraint based on age, height, and/or weight. Two nationally certified child passenger safety technicians and injury prevention specialists separately reviewed patient information. As child passenger safety technicians with the technical experience and knowledge on proper car seat use and installation, they made recommendations on proper restraint type for each study patient and appropriateness of the documented restraint used. Appropriateness was determined by current age-based safety restraint laws and best practices. While child safety restraint laws may vary by state,^16^ current best practice guidelines from the American Academy of Pediatrics (AAP) are as follows: (1) use of rear-facing car seats until they are outgrown, (2) use of forward-facing car seats through at least 4 years of age, (3) booster seats through minimum 8 years of age, (4) seat belts (including lap and shoulder belts) for all children who have outgrown booster seats, and (5) placement in the rear seat of the vehicle for all children <13 years.^17^

### The Childhood Opportunity Index 2.0

The COI is a verified multidimensional metric that uses US census tract and zip code data to reflect neighborhood conditions that contribute to healthy childhood development.^13–15^ In the present study, COI was assigned at the zip code level. The COI is based on 29 neighborhood-level indicators that represent access to resources in three domains that are known to be important for childhood development: education, health and environment, and socioeconomic. Examples of neighborhood-level indicators include but are not limited to educational and social resources, presence of early childhood education centers, health insurance coverage, toxic exposures, neighborhood walkability, household income, and employment rate.^15 18^ Neighborhood indicators are weighed based on their degree of association with health and socioeconomic outcomes. Indicator scores are converted into index z-scores (1–100) and then expressed as five different levels of opportunity: very low, low, moderate, high, and very high.^14 18 19^ The COI has a wide range of applications and has been used in research investigating childhood resource inequities and the impact of child opportunity on child health.^18^

### Statistical analysis

We described demographic and clinical characteristics using frequencies and percentages for categorical variables, and median and interquartile range (IQR) were used to describe continuous variables. Χ^2^ and Kruskal-Wallis tests were used to evaluate variables of interest and safety restraint use. Multivariable logistic regression analysis controlling for age was performed to identify associations between COI and appropriate safety restraint use. Covariates of race, ethnicity, and insurance type were evaluated for confounding but did not impact the primary exposure of interest nor were they significantly associated with safety restraint on their own, therefore, they were not included in the final model. Additionally, health insurance is an indicator under the health and environment domain of calculating the COI, carrying the largest weighted index of 0.19.^14^ A sensitivity analysis was conducted to remove adolescents of driving age who were the drivers in the MVCs (online supplemental table 1). Additional multivariable regression analyses were performed for children aged <8 years and ≥8 years, with the age cut-off based on California Highway Patrol guidelines on the minimum age requiring additional safety restraint devices rather than seat belts alone.^20^ All statistical analyses were two sided with a *p* value <0.05 considered significant. Analysis was performed using SAS software V.9.4 (SAS Institute). Findings were reported in concordance with the Strengthening the Reporting of Observational Studies in Epidemiology reporting guideline for cross-sectional studies.^21^ Due to the retrospective nature of this study, patients or the public were not involved in the development or analysis of this study.

10.1136/wjps-2023-000703.supp1 Supplementary data

## Results

### Study sample characteristics

**Figure 1:**
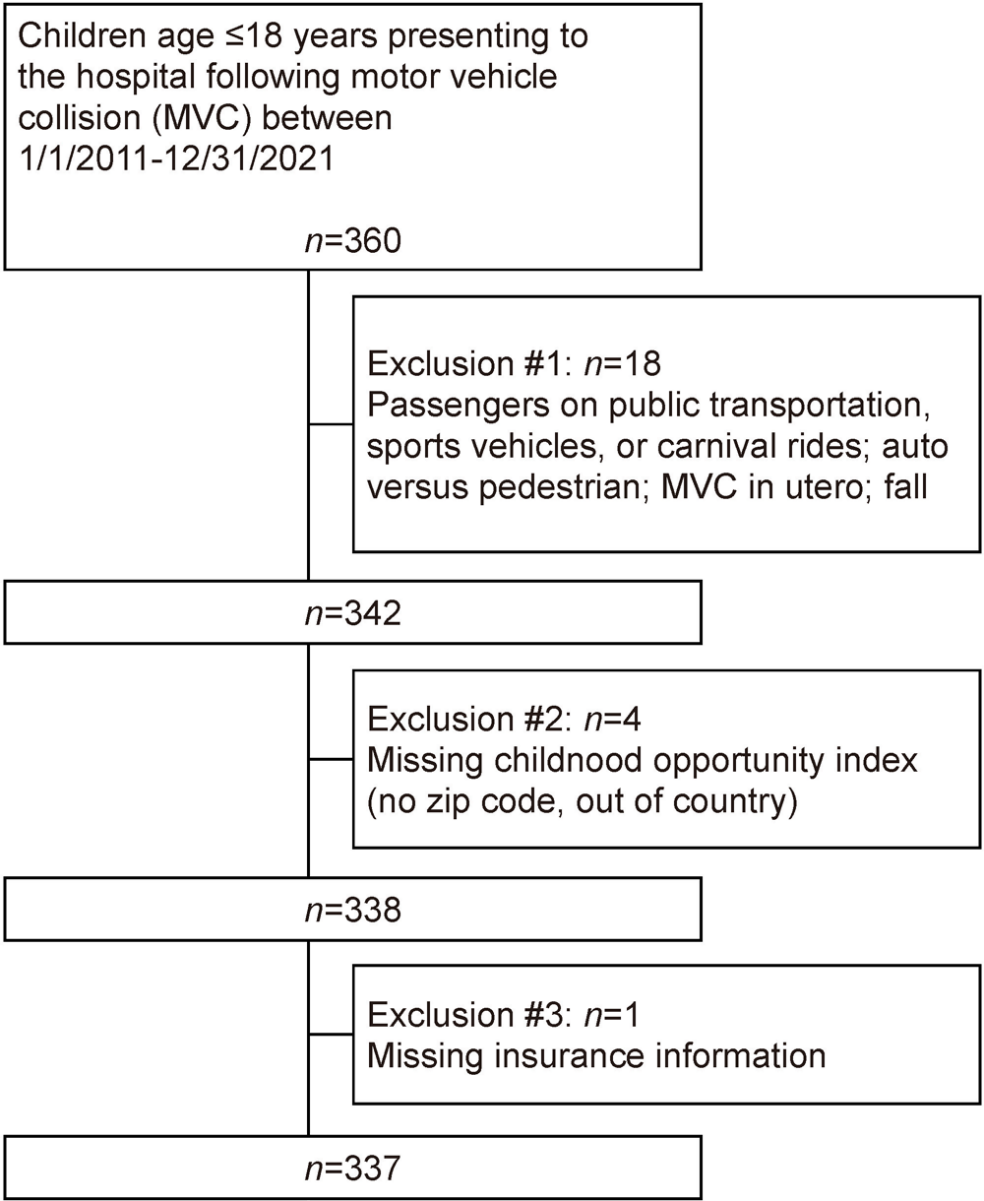
Patient cohort selection.

Overall, 360 patients who presented to our hospital following MVCs between January 1, 2011 and December 31, 2021 were identified (figure 1).

The analytic cohort consisted of 337 patients: 165 (49.0%) were female, with a median age of 4.0 years (IQR 1.7–7.0 years) (table 1). Appropriate safety restraint use was identified in 249/337 (73.9%) children/adolescents, while 88/337 (26.1%) children/adolescents were determined to not be appropriately restrained (73 non-restrained, 15 improperly restrained) (table 1). Safety restraint types were infant and child car seats (*n*=179, 53.1%), booster seats (*n*=60, 17.8%), lap belts (*n*=1, 0.3%), and seat belts (*n*=25, 7.4%). Overall, most children/adolescents were from very low-COI neighborhoods (*n*=143, 42.4%), followed by low-COI (*n*=75, 22.3%), moderate-COI (*n*=61, 18.1%), high-COI (*n*=40, 11.9%), and very high-COI (*n*=18, 5.3%) neighborhoods (table 1).

**Table 1:** Demographics of the patient cohort overall and stratified into appropriately restrained and not appropriately restrained (including non-restrained and improperly restrained children)

Clinical characteristic	Overall *n*=337	Not appropriately restrained *n*=88	Appropriately restrained *n*=249	*P* value
Sex, *n* (%)				0.635
Female	165 (49.0)	45 (51.1)	120 (48.2)	
Male	172 (51.0)	43 (48.9)	129 (51.8)	
Age (years), median (IQR)	4 (1.7–7.0)	8 (4.0–14.0)	3 (1.1–5.0)	<0.001
Weight (kg), median (IQR)*	17 (11.0–27.0)	33 (18.0–57.0)	15.0 (9.2–21.5)	<0.001
Race/ethnicity, *n* (%)				0.158
Asian	8 (2.4)	1 (1.1)	7 (2.8)	
Black	53 (15.7)	19 (21.6)	34 (13.7)	
Other	88 (26.1)	27 (30.7)	61 (24.5)	
White	30 (8.9)	5 (5.7)	25 (10.0)	
Hispanic	158 (46.9)	36 (40.9)	122 (49.0)	
Primary language, *n* (%)				0.616
English	263 (78.0)	67 (76.1)	196 (78.7)	
Non-English	74 (22.0)	21 (23.9)	53 (21.3)	
Insurance type, *n* (%)				0.421
Public	250 (74.2)	69 (78.4)	181 (72.7)	
Private	53 (15.7)	10 (11.4)	43 (17.3)	
Other	34 (10.1)	9 (10.2)	25 (10.0)	
Injury Severity Score**†**				0.007
Median (IQR)	4 (1–10)	5 (2–14)	2 (1–10)	
Minimum	1	1	1	
Maximum	75	75	75	
≥15, *n* (%)	42 (17.5)	16 (18.4)	26 (10.4)	0.292
Restraint type, *n* (%)				<0.001
Car seat	179 (53.1)	4 (4.6)	175 (70.3)	
Booster seat	60 (17.8)	5 (5.7)	55 (22.1)	
Lap belt	1 (0.3)	1 (1.1)	0 (0.0)	
Seat belt	25 (7.4)	6 (6.8)	19 (7.6)	
None	72 (21.4)	72 (81.8)	0 (0.0)	
Childhood Opportunity Index (COI), *n* (%)				0.031
Very low	143 (42.4)	33 (37.5)	110 (44.2)	
Low	75 (22.3)	23 (26.1)	52 (20.9)	
Moderate	61 (18.1)	10 (11.4)	51 (20.5)	
High	40 (11.9)	13 (14.8)	27 (10.8)	
Very high	18 (5.3)	9 (10.2)	9 (3.6)	

*n=323.

†*n*=240.

### Restraint use

Not appropriately restrained patients were older (median age 8.0 years vs 3.0 years; *p*<0.001) and of greater weight (median 33.0 kg vs 15.0 kg; *p*<0.001) than appropriately restrained patients, with no difference in sex (*p*=0.635), race/ethnicity (*p*=0.158), or insurance type (*p*=0.421). Not appropriately restrained children/adolescents had significantly higher median ISS (5; IQR 2–14) compared with appropriately restrained children/adolescents (2; IQR 1–10) (*p*=0.007), although the two groups did not differ based on number of patients with severe polytrauma, as indicated by an ISS≥15 (18.4% vs 10.4%, *p*=0.292).^22^ More not appropriately restrained patients than appropriately restrained patients were from low-COI (26.1% vs 20.9%), high-COI (14.8% vs 10.8%), and very high-COI (10.2% vs 3.6%) neighborhoods, while more appropriately restrained patients were from very low-COI (44.2% vs 37.5%) and moderate-COI (20.5% vs 11.4%) neighborhoods (*p*=0.031) (table 1). A sensitivity analysis performed for adolescents who were the drivers at the time of MVCs demonstrated similar safety restraint use.

**Figure 2:**
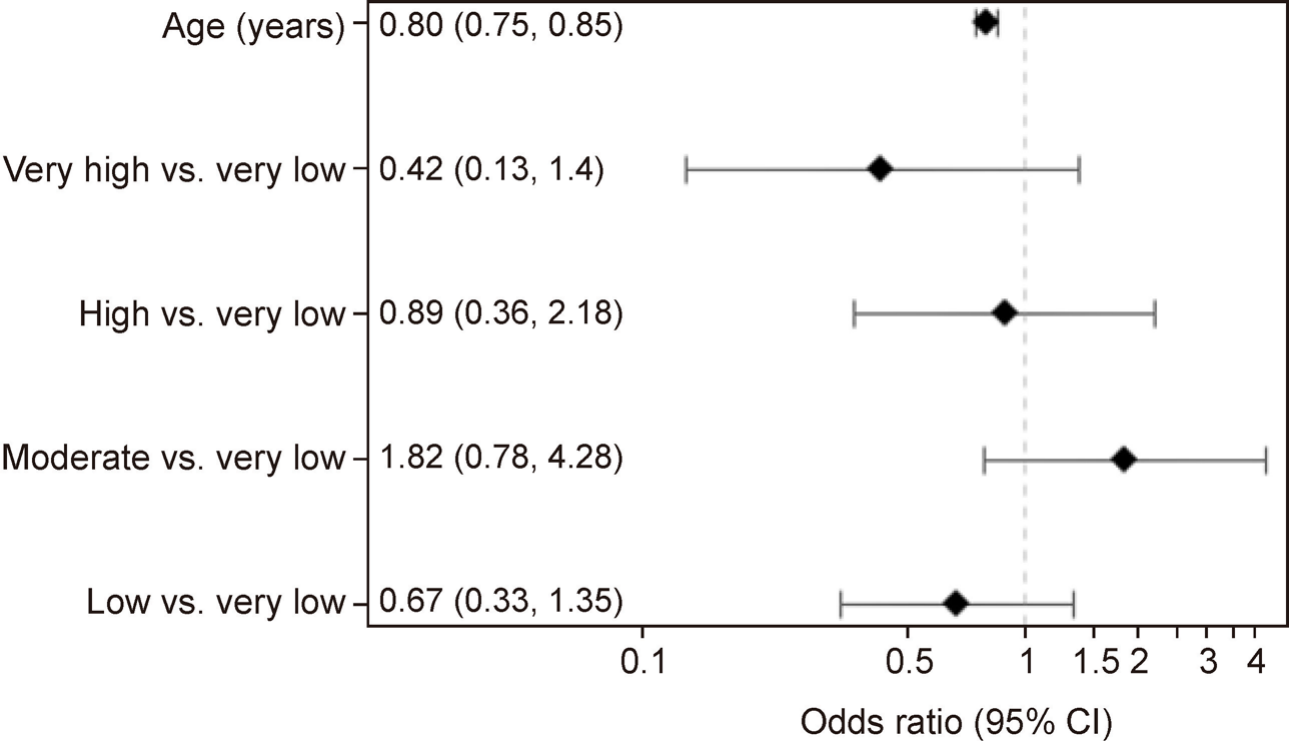
Association between Childhood Opportunity Index (COI) and appropriate safety restraint use. Multivariable logistic regression analysis for appropriate safety restraint use based on COI levels (reference: very low COI), controlling for age.

On multivariable regression controlling for age, there were no significant associations between appropriate restraint use and COI level. Children/adolescents from moderate-COI neighborhoods had a higher OR, although not significant, of being appropriately restrained compared with children/adolescents from very low-COI neighborhoods (OR=1.82, 95% CI 0.78, 4.28) (figure 2). There was no significant association between COI level and being appropriately restrained, as compared with children/adolescents from very low-COI neighborhoods, children/adolescents from low-COI, high-COI, and very high-COI neighborhoods had ORs of 0.67 (95% CI 0.33, 1.35), 0.89 (95% CI 0.36, 2.18), and 0.42 (95% CI 0.13, 1.40), respectively. Age was associated with appropriate safety restraint use, with older children/adolescents less likely to be appropriately restrained (OR=0.80, 95% CI 0.75, 0.85) (figure 2).

In an additional subset analysis of children stratified based on age <8 years and ≥8 years adjusting for ethnicity and insurance type, younger children aged <8 years were less likely to be appropriately restrained compared with older children (table 2). The same analysis that included only children aged <8 years did not demonstrate an association between insurance type and appropriate safety restraint use (table 3). In both models, there were no significant associations between COI level and appropriate safety restraint use (tables 2 and 3).

**Table 2:** Multivariable regression analysis of appropriate safety restraint use based on age category (<8 years vs ≥8 years), adjusting for Hispanic ethnicity and public insurance type

Effect	OR	95% CI	*P* value
COI (vs very low)			
Low	0.60	0.30 to 1.21	0.15
Moderate	1.96	0.81 to 4.75	0.14
High	0.79	0.33 to 1.91	0.60
Very high	0.226	0.06 to 0.80	0.02
Age (<8 years vs ≥8 years)	0.11	0.06 to 0.20	<0.0001
Ethnicity (Hispanic vs non-Hispanic)	1.40	0.79 to 2.50	0.25
Insurance (vs public)			
Private	2.91	1.15 to 7.38	0.03
Other	1.60	0.62 to 4.15	0.34

COI, Childhood Opportunity Index.

**Table 3:** Stratified multivariable regression analysis of appropriate safety restraint use based on age groups <8 years and ≥8 years, adjusting for Hispanic ethnicity and public insurance type

Effect	OR	95% CI	*P* value
**Age <8 years**			
COI (vs very low)			
Low	0.80	0.35 to 1.84	0.60
Moderate	2.67	0.72 to 9.89	0.14
High	0.67	0.21 to 2.08	0.48
Very high	0.32	0.07 to 1.42	0.13
Ethnicity (Hispanic vs non-Hispanic)	1.02	0.50 to 2.08	0.96
Age (years)	0.79	0.68 to 0.92	<0.01
Insurance (vs public)			
Private	1.45	0.48 to 4.45	0.51
Other	1.48	0.32 to 6.85	0.62
**Age ≥8 years**			
COI (vs very low)			
Low	0.31	0.06 to 1.60	0.16
Moderate	1.79	0.42 to 7.58	0.43
High	0.91	0.19 to 4.31	0.90
Very high	0.29	0.02 to 4.49	0.38
Ethnicity (Hispanic vs non-Hispanic)	3.05	0.90 to 10.36	0.07
Age (years)	0.85	0.71 to 1.03	0.09
Insurance (vs public)			
Private	12.27	2.21 to 68.06	<0.01
Other	2.37	0.57 to 9.83	0.24

COI, Childhood Opportunity Index.

## Discussion

We sought to describe variations in the use of child/adolescent motor vehicle safety restraints among patients presenting to our trauma center following MVCs. The results of our study demonstrate a large number of children/adolescents who are not appropriately restrained at time of MVC. Additionally, safety restraint use is not limited to only families from neighborhoods with low resources, as indicated by different neighborhood COIs. Our study is also one of the first to use neighborhood COI based on home zip codes to identify potential disparities in motor vehicle safety restraint use.

Child safety restraints are known to reduce morbidity and mortality risk in children.^1 6–8^ Improper child motor vehicle restraint use is associated with greater need for trauma activations on arrival to the ED, injury severity, and mortality risk.^6–8^ Compared with seat belts, age-appropriate child safety restraints have been reported to reduce risk of injury by up to 82% and reduce risk of death by almost 30%.^17 23 24^ The AAP strongly supports routine assessment and education for families with children regarding proper child safety in motor vehicles.^17^

Disparities exist in child safety restraint use and outcomes of children in MVCs. According to the Centers for Disease Control and Prevention, rates of unrestrained child passenger deaths after MVC are higher in black and Hispanic children compared with white children.^5^ In a large study of over 1200 children in Cincinnati, Rangel *et al* demonstrated that black children compared with white children were less likely to be restrained or properly restrained, with the greatest difference observed in use of car seats.^11^ They also reported that children with public insurance were less likely to be in proper child safety restraints than those privately insured.^12^ In contrast, Sylvester *et al* did not identify significant differences in sex, race, or low-income status between non-restrained, improperly restrained, and properly restrained children in Florida.^9^ A plausible reason for the discrepancies between these studies may be that they represent two different geographic regions with differing patient populations, public health resources, and state car seat laws. Our study is unique in that it represents a different population of patients from Los Angeles. Furthermore, rather than focusing on limited variables of race, ethnicity, or insurance type, we describe disparities in safety restraint use among children in MVCs using a more comprehensive metric of the COI.^18 25–27^ The COI has been mostly used in the context of general pediatrics such as childhood obesity and pediatric emergency care utilization.^19 28 29^ Few studies exist using the COI to investigate disparities in pediatric surgery. Recently, Bouchard *et al* linked data from the Pediatric Health Information Systems database to the COI 2.0 database to demonstrate differences in risk of complicated appendicitis.^15^ To our knowledge, our study is one of the first to use the COI to investigate variations in social determinants of health in pediatric trauma patients.

Our study demonstrates differences in motor vehicle child safety restraint use among children/adolescents from neighborhoods with different levels of childhood opportunity, although the analysis did not demonstrate any significant associations between COI and appropriate safety restraint use. The broad range in CIs suggests lack of precision, which may be due to a small sample size. Contrary to our hypothesis, most appropriately restrained children/adolescents were from neighborhoods with very low COI, while not appropriately restrained children/adolescents were mostly from low-COI and moderate-COI neighborhoods. Additionally, there were more patients from high-COI and very high-COI neighborhoods in the not appropriately restrained group than appropriately restrained group. One possible explanation is that private vehicles may be more readily available to families and children from higher COI neighborhoods, including older children, adolescents, and teenagers who may have been driving at the time of the MVC. These findings can also be supported by previous work from Shinar *et al* where income and level of education did not accurately predict adherence to safe motor vehicle practices such as safety belt use.^30^ Although an at-risk population based on COI was not identified, our study suggests that children with private insurance are more likely to be appropriately restrained (table 3), thus suggesting that perhaps publicly insured children/families may benefit from targeted injury prevention initiatives.

Despite current education and counseling of parents on child safety restraint use, an alarmingly high number of children continue to be non-restrained or improperly restrained in motor vehicles.^9 12 31^ Injury prevention programs are crucial aspects of pediatric trauma systems and encompass both education and intervention. Muller *et al* previously demonstrated that car seat classes at our institution improve parent knowledge and awareness through a comprehensive, multifaceted curriculum including hands-on practice.^32^ In settings where in-person resources are limited, virtual classes also improve caregiver proficiency in child safety restraints.^33^ However, education alone may not be sufficient to increase appropriate motor vehicle child safety restraint use. A prospective study by Gittelman *et al* found that parents provided with booster seat education prior to discharge from the ED were less likely to purchase and use booster seats, while the majority of families who were provided both teaching and free booster seat installation reported consistent use of booster seats for their children.^34^ Similarly, Apsler *et al* improved child safety restraint use by providing education and free booster seats to day care families, while financial initiatives and policy changes did not result in meaningful change.^35^ These studies highlight the importance of resources in improving child safety restraint use, regardless of neighborhood COI. Within our institution, the Injury Prevention Program provides education and resources as well as free inpatient and outpatient services to families in the community including, but not limited to, hands-on, personalized car seat classes with installations and inspections, special needs evaluations, and permanent car seats to families receiving state or county public assistance. Potential methods to improve adherence to safety restraint guidelines by families include providing education and resources in the families’ primary language, offering follow-up appointments following the initial intervention (bedside education, car seat class attendance), and providing appropriate car seats. Interventions should also be offered to families presenting to the ED or inpatient setting for any reason, rather than only those who present following MVCs.^36^

This study is not without limitations. Car seat orientation, whether forward facing or rear facing, was unable to be determined for every case based on the information in the trauma database and the lack of specificity in medical provider documentation. While car seat placement is specified in safety restraint guidelines, we based our criteria of appropriateness of car seat use on age and weight or height, when available.^17^ Use of restraint was determined based on information gathered from law enforcement and EMS responders, patients, or patients’ caregivers. Due to the retrospective nature of the study, it was impossible for us to physically verify the specific restraint types, and thus non-differential misclassification may have contributed to the results of this study.^37^ However, any error in EMS, ED, or caregiver reports would not be systematic and thus would not favor a specific outcome. Additionally, it is not the usual practice for medical staff to physically assess patient restraint types when they present to the hospital or ED. Hospital staff are not the first point of contact with children involved in MVCs and are reliant on those reporting from the scene of the collision. For example, although a car seat is present and use is reported, technical use may be inappropriate such that the harness height adjustment may be incorrect or may have not been buckled properly. Thus, it is possible that more patients were improperly restrained than reported. Although some patients were noted in the documentation to have been using the appropriate restraint in an improper manner (and thus were categorized as not appropriately restrained), it is possible that patients in this category were missed because it was not elucidated during patient assessment or documented in patient records. Additionally, because several patients in our study resided in neighborhoods with zip codes outside of Los Angeles County, our analysis of COI is not exclusive to Los Angeles neighborhoods. Lastly, the findings of this study represent a sample of patients at a single institution and thus may be biased and not reflect national trends from random sampling.

Despite its limitations, our study provides evidence that lack of proper safety restraint use is not exclusive to families from low-COI neighborhoods and that disparities exist between children/adolescents with varying levels of access to resources. Neighborhood COI may be helpful in identifying patient populations that should be targeted by safety restraint education and injury prevention initiatives. Furthermore, this is the first study in pediatric surgical literature that specifically attempts to describe disparities among pediatric trauma patients using the COI. The COI may represent an interesting and valuable tool for future studies in pediatric trauma.

In conclusion, appropriate child motor vehicle safety restraint use varies among children and adolescents from neighborhoods with different resources, as indicated by COI. This study introduces populations of families who may benefit from targeted education and interventions to reduce injury and death from MVCs.

## Data Availability

Data are available upon reasonable request.

## References

[1] CDC . Injury center - motor vehicle crashes: a leading cause of death for children. 2021. Available: https://www.cdc.gov/transportationsafety/child_passenger_safety/cps-factsheet.html#References [Accessed 9 Feb 2022].

[2] Centers for Disease Control and Prevention . Centers for disease control and prevention’s web-based injury Statistics query and reporting system. Query and Reporting System. Available: https://webappa.cdc.gov/sasweb/ncipc/leadcause.html [Accessed 9 Feb 2022].

[3] National Center for Statistics and Analysis. (2022, March - revised) . Data: children 2019 (traffic safety facts. report no. DOT HS 813 122). National Highway Traffic Safety Administration. Available: https://crashstats.nhtsa.dot.gov/Api/Public/ViewPublication/813122 [accessed 9 Feb 2022]

[4] Nance ML , Lutz N , Arbogast KB , et al . Optimal restraint reduces the risk of abdominal injury in children involved in motor vehicle crashes. Ann Surg 2004;239:127–31. 10.1097/01.sla.0000103068.51037.20 14685110 PMC1356202

[5] Sauber-Schatz EK , West BA , Bergen G , et al . Vital signs: restraint use and motor vehicle occupant death rates among children aged 0-12 years - United States, 2002-2011. MMWR Morb Mortal Wkly Rep 2014;63:113–8. 24500292 PMC4584642

[6] Wolf LL , Chowdhury R , Tweed J , et al . Factors associated with pediatric mortality from motor vehicle crashes in the United States: a state-based analysis. J Pediatr 2017;187:295–302. 10.1016/j.jpeds.2017.04.044 28552450 PMC5558848

[7] Urrechaga EM , Cioci AC , Allen MK , et al . Improper restraint use in pediatric patients involved in motor vehicle collisions. J Surg Res 2022;273:57–63. 10.1016/j.jss.2021.12.015 35030430

[8] Findlay BL , Melucci A , Dombrovskiy V , et al . Children after motor vehicle crashes: restraint utilization and injury severity. J Pediatr Surg 2019;54:1411–5. 10.1016/j.jpedsurg.2018.10.046 30446393

[9] Sylvester S , Schwartz JM , Hsu A , et al . Pediatric safety restraint use in motor vehicle crashes at a level I safety-Net trauma center. J Surg Res 2021;258:132–6. 10.1016/j.jss.2020.08.053 33010558

[10] Roehler DR , Elliott MR , Quinlan KP , et al . Factors associated with unrestrained young passengers in motor vehicle crashes. Pediatrics 2019;143. 10.1542/peds.2018-2507 30718381

[11] Falcone RA , Brown RL , Garcia VF . The epidemiology of infant injuries and alarming health disparities. J Pediatr Surg 2007;42:172–6. 10.1016/j.jpedsurg.2006.09.015 17208560

[12] Rangel SJ , Martin CA , Brown RL , et al . Alarming trends in the improper use of motor vehicle restraints in children: implications for public policy and the development of race-based strategies for improving compliance. J Pediatr Surg 2008;43:200–7. 10.1016/j.jpedsurg.2007.09.045 18206483

[13] diversitydatakids.org . Child opportunity index 2.0 database 2022. 2022. Available: https://data.diversitydatakids.org/dataset/coi20-child-opportunity-index-2-0-database?_external=True [Accessed 9 Feb 2022].

[14] Noelke C , Noelke C , Mcardle N . Child opportunity index 2.0 technical documentation child opportunity index 2.0 technical documentation. 2020. Available: diversitydatakids.org/research-library/research-brief/how-we-built-it [accessed 9 Feb 2022]

[15] Bouchard ME , Kan K , Tian Y , et al . Association between neighborhood-level social determinants of health and access to pediatric Appendicitis care. JAMA Netw Open 2022;5:e2148865. 10.1001/jamanetworkopen.2021.48865 35171257 PMC8851303

[16] Bae JY , Anderson E , Silver D , et al . Child passenger safety laws in the United States, 1978-2010: policy diffusion in the absence of strong Federal intervention. Soc Sci Med 2014;100:30–7. 10.1016/j.socscimed.2013.10.035 24444836 PMC3899584

[17] Durbin DR , Hoffman BD , COUNCIL ON INJURY, VIOLENCE, AND POISON PREVENTION . Child passenger safety. Pediatrics 2018;142:e20182461. 10.1542/peds.2018-2461 30166367

[18] Acevedo-Garcia D , Noelke C , McArdle N , et al . Racial and ethnic inequities in children’s neighborhoods: evidence from the new child opportunity index 2.0. Health Aff (Millwood) 2020;39:1693–701. 10.1377/hlthaff.2020.00735 33017244

[19] Bettenhausen JL , Noelke C , Ressler RW , et al . The Association of the childhood opportunity index on pediatric Readmissions and emergency Department Revisits. Acad Pediatr 2022;22:614–21. 10.1016/j.acap.2021.12.015 34929386 PMC9169565

[20] California Highway Patrol . Child safety seats. 2017. Available: www.chp.ca.gov/programs-services/programs/child-safety-seats#:~:text=California%20Law&text=%E2%80%8BChildren%20under%20the%20age,secured%20by%20a%20safety%20belt [Accessed 9 Feb 2022].

[21] Vandenbroucke JP , von Elm E , Altman DG , et al . Strengthening the reporting of observational studies in epidemiology (STROBE): explanation and elaboration. PLoS Med 2007;4:e297. 10.1371/journal.pmed.0040297 17941715 PMC2020496

[22] Brown JB , Gestring ML , Leeper CM , et al . The value of the injury severity score in pediatric trauma: time for a new definition of severe injury? J Trauma Acute Care Surg 2017;82:995–1001. 10.1097/TA.0000000000001440 28328674 PMC5464600

[23] Arbogast KB , Durbin DR , Cornejo RA , et al . An evaluation of the effectiveness of forward facing child restraint systems. Accid Anal Prev 2004;36:585–9. 10.1016/S0001-4575(03)00065-4 15094411

[24] Elliott MR , Kallan MJ , Durbin DR , et al . Effectiveness of child safety seats vs seat belts in reducing risk for death in children in passenger vehicle crashes. Arch Pediatr Adolesc Med 2006;160:617–21. 10.1001/archpedi.160.6.617 16754824

[25] Kolak M , Bhatt J , Park YH , et al . Quantification of neighborhood-level social determinants of health in the Continental United States. JAMA Netw Open 2020;3:e1919928. 10.1001/jamanetworkopen.2019.19928 31995211 PMC6991288

[26] Singh GK . Area deprivation and widening inequalities in US mortality, 1969–1998. Am J Public Health 2003;93:1137–43. 10.2105/ajph.93.7.1137 12835199 PMC1447923

[27] Fuller-Rowell TE , Nichols OI , Jokela M , et al . A changing landscape of health opportunity in the United States: increases in the strength of association between childhood socioeconomic disadvantage and adult health between the 1990s and the 2010S. Am J Epidemiol 2021;190:2284–93. 10.1093/aje/kwab060 33710274 PMC8799901

[28] Aris IM , Rifas-Shiman SL , Jimenez MP , et al . Neighborhood child opportunity index and adolescent Cardiometabolic risk. Pediatrics 2021;147:e2020018903. 10.1542/peds.2020-018903 33479165 PMC7906069

[29] Fritz CQ , Fleegler EW , DeSouza H , et al . Child opportunity index and changes in pediatric acute care utilization in the COVID-19 pandemic. Pediatrics 2022;149:e2021053706. 10.1542/peds.2021-053706 35233618

[30] Shinar D , Schechtman E , Compton R . Self-reports of safe driving behaviors in relationship to sex, age, education and income in the US adult driving population. Accid Anal Prev 2001;33:111–6. 10.1016/s0001-4575(00)00021-x 11189114

[31] Macy ML , Cunningham RM , Resnicow K , et al . Disparities in age-appropriate child passenger restraint use among children aged 1 to 12 years. Pediatrics 2014;133:262–71. 10.1542/peds.2013-1908 24420814 PMC3904276

[32] Muller VM , Burke RV , Arbogast H , et al . Evaluation of a child passenger safety class in increasing parental knowledge. Accid Anal Prev 2014;63:37–40. 10.1016/j.aap.2013.10.021 24252556

[33] Kuroiwa E , Ragar RL , Langlais CS , et al . Car seat education: a randomized controlled trial of teaching methods. Injury 2018;49:1272–7. 10.1016/j.injury.2018.05.003 29739654

[34] Gittelman MA , Pomerantz WJ , Laurence S . An emergency Department intervention to increase booster seat use for lower socioeconomic families. Acad Emerg Med 2006;13:396–400. 10.1197/j.aem.2005.11.002 16531596

[35] Apsler R , Formica SW , Rosenthal AF , et al . Increases in booster seat use among children of low income families and variation with age. Inj Prev 2003;9:322–5. 10.1136/ip.9.4.322 14693893 PMC1731035

[36] Macy ML , Kandasamy D , Resnicow K , et al . Pilot trial of an emergency Department-based intervention to promote child passenger safety best practices. Acad Emerg Med 2019;26:770–83. 10.1111/acem.13687 30637887 PMC6626697

[37] Brennan AT , Getz KD , Brooks DR , et al . An underappreciated misclassification mechanism: implications of nondifferential dependent misclassification of covariate and exposure. Annals of Epidemiology 2021;58:104–23. 10.1016/j.annepidem.2021.02.007 33621629

