# Supplementary material for: Childhood opportunity and appropriate use of child safety restraints in motor vehicle collisions
Source: World J Pediatr Surg. 2024 Apr 2;7(2):e000703. doi: 10.1136/wjps-2023-000703 (PMC10989117; doi:10.1136/wjps-2023-000703)
Supplement: Supplementary data [file wjps-2023-000703supp001.pdf]

Supplementary Table 1. Odds ratios from sensitivity analysis excluding adolescents of driving age (n=7).

Effect	Estimate	95% Confidence Limits	p-value
**COI (vs. Very Low)**			
Low	0.680	0.336-1.380	0.286
Moderate	2.044	0.856-4.881	0.107
High	1.037	0.405-2.652	0.940
Very High	0.494	0.139-1.762	0.277
**Age (Years)**	0.783	0.732-0.837	<0.0001
